# Determining travel fluxes in epidemic areas

**DOI:** 10.1371/journal.pcbi.1009473

**Published:** 2021-10-27

**Authors:** Daipeng Chen, Yuyi Xue, Yanni Xiao

**Affiliations:** 1 School of Mathematics and Statistics, Xi’an Jiaotong University, Xi’an, China; 2 Mathematical Institute, Leiden University, Leiden, The Netherlands; The Pennsylvania State University, UNITED STATES

## Abstract

Infectious diseases attack humans from time to time and threaten the lives and survival of people all around the world. An important strategy to prevent the spatial spread of infectious diseases is to restrict population travel. With the reduction of the epidemic situation, when and where travel restrictions can be lifted, and how to organize orderly movement patterns become critical and fall within the scope of this study. We define a novel diffusion distance derived from the estimated mobility network, based on which we provide a general model to describe the spatiotemporal spread of infectious diseases with a random diffusion process and a deterministic drift process of the population. We consequently develop a multi-source data fusion method to determine the population flow in epidemic areas. In this method, we first select available subregions in epidemic areas, and then provide solutions to initiate new travel flux among these subregions. To verify our model and method, we analyze the multi-source data from mainland China and obtain a new travel flux triggering scheme in the selected 29 cities with the most active population movements in mainland China. The testable predictions in these selected cities show that reopening the borders in accordance with our proposed travel flux will not cause a second outbreak of COVID-19 in these cities. The finding provides a methodology of re-triggering travel flux during the weakening spread stage of the epidemic.

## Author summary

Human infectious diseases spread from their origins to other places with population movements. In order to curb the spatial spread of infectious diseases, many countries and regions may introduce some travel restrictions when the epidemic is severe, and reopen the borders as the epidemic eases. This process involves some important issues such as the start and end time of travel restrictions, the geographical scope of the implementation of the exit strategy, and the allowable passenger flow on traffic lines. Here, we integrate multi-source data with a mathematical model, and consequently develop a new method to determine the travel flux in epidemic areas. As an application, we use this method to calculate when and where the travel restrictions targeting COVID-19 in China in early 2020 could be lifted, and how to optimize passenger flow along the traffic lines among the reopened cities. The testable predictions indicate that the population flow in accordance with our proposed movement pattern will not cause a resurgent outbreak of COVID-19 in the cities studied.

## Introduction

In December 2019, the first COVID-19 case caused by infection with the coronavirus SARS-CoV-2 was reported in Wuhan [1]. The coronavirus then quickly spread out nationally [2] and internationally [3]. To prevent the spread of the novel virus, unprecedented measures such as travel restriction, quarantine and isolation followed by contact tracing were introduced by the Chinese government [4]. Most notably, strict travel restrictions were imposed on Wuhan and nearby cities from 23 January 2020. Subsequently, the highest level of alert and responses to the public health emergency were activated in mainland China [4]. The restrictions on mobility were also imposed on many other countries around the world to mitigate the spatial spread of SARS-CoV-2 [5]. Several studies have evaluated the effect of travel restrictions on the spread of SARS-CoV-2 [6] and have demonstrated that the lockdown imposed on Wuhan combined with measures to control movements in and out of the city reduced the number of domestic [7] and international [8] infections.

In epidemiology, a term (cordon sanitaire) refers to the restrictions of movement of people into or out of a specific area such as a community, city or country [9]. In addition to responding to COVID-19 [10], cordons sanitaire have also been established to stop the spread of other infectious diseases such as the 2001 foot and mouth disease epidemic in Britain [11], the 2003 SARS epidemic in China [12], the 2009 H1N1 flu epidemic in Mexico [13] and the 2014 Ebola epidemic in West Africa [14]. Reduced mobility between areas plays a potentially important role in decreasing transmission [15, 16], but it also has a potential social and economic impact [17, 18]. Once infectious disease or diseases are under control or successfully contained in some specific areas, the reopening of borders has been put on the agenda. However, it is not clear how to balance the trade-offs between inter-regional mobility and the risk of potential new outbreaks. Determining where and when restrictions on mobility can be relaxed and the design of a new orderly movement pattern fall within the scope of this study.

In the most extreme case, the cordon is not lifted until the infectious disease is extinguished [19], but it is not suitable for curbing a large-scale emergent epidemic [20], and hence many countries are facing a balance between reopening and preventing the epidemic from rebounding. A number of studies formulated mathematical models including mobility to investigate the effect of massive movements on new infections [21–24]. However, few studies have focused on the trade-off between mobility restrictions and virus spread. Preparing for a responsible lockdown exit strategy [25] has been extremely important not only for the ongoing COVID-19 epidemic [26], but also for future pandemic outbreaks. Most integrated studies on mobility data and the spatial spread of infectious diseases are based on meta-population models [27]. Combining the model with mobility data, Linka et al. [28] provided an exit strategy for the travel ban in Canada. In their study, they made reopening forecasts by updating the fraction by which the baseline movement matrix was multiplied and running the model repeatedly. Once some acceptable simulations were obtained, the corresponding movement matrixes were translated into suggestions to guide population mobility. Based on the same model, Ruktanonchai et al. [29] assessed the impact of coordinated COVID-19 exit strategies across Europe. Similar to the work of Linka et al. [28], they simulated their model under different parameter sets and initial states and found that appropriate coordinated exit strategies greatly improved the possibility of curbing the spread of COVID-19 in Europe.

Although meta-population models have provided a rich and nuanced perspective on predicting epidemic spread [30] and evaluating the effectiveness of interventions on mobility [31], they would become highly complex and difficult to compute if more spatial heterogeneity was introduced [26]. Another limitation of the previous studies is that the methods were based on adjusting parameters or updating initial states. This approach generally only provides some suggestions on the order [29] or degree [28] of reopening in some epidemic areas, and does not provide relatively detailed information on where and when the restrictions on mobility can be relaxed, nor answer what kind of population movement pattern among various areas is optimal. To our best knowledge, there is just one study [32] in which the authors developed a method to determine the optimal travel flux in epidemic areas and applied their method in the Netherlands. Given the time horizon and regional division, Gösgens et al. [32] proposed an optimal function which was positively related to mobility and negatively related to infections. However, their method is computationally too expensive because it involves updating regional divisions and solving a meta-population epidemiological model.

To solve the difficulties induced by the meta-population model, by defining a diffusion distance, we have reduced the complex spatiotemporal spread of virus into a simple wave mode, and formulated a reaction diffusion equation. In this study, we further extend the reaction diffusion system to a reaction diffusion-drift equation, where the drift term corresponds to deterministic travel fluxes. Instead of testing different movement patterns, here we develop a novel multi-source data fusion method that can integrate the model with data to calculate the optimal travel flux in epidemic areas. Unlike the work of Gösgens et al. [32], our objective function and constraints give a convex optimization, which means that the global optimum is available. As a case study, we test this method with data for mainland China where a strict lockdown was implemented in early 2020. We can thus obtain the specific timings for reopening, select suitable cities and determine the travel fluxes among these cities that would allow them to reopen as well as keeping the epidemic from rebounding.

## General methods

### Diffusion distance

The complexity of human mobility, especially via air traffic and high-speed train, has reconstructed subregions around the world into a high-dimensional spatial structure, which makes it increasingly difficult to model the spread of emerging infectious diseases. To describe the quick spread of infectious diseases over long distances using a simple reaction-diffusion system [33], we define the “diffusion distance” based on the mobility network (MN) among various locations. The mobility network (MN) [34] is a directed weighted network, and the weights of the edges between pairs of nodes (locations) are the relative population flow among them. It is very important to note that the absolute passenger flux between two locations is not important in this study. What we want to characterize is that the traffic has made a new ranking of the connections among different cities. Let (X,A,μ) be a measure space, *W*(*x*, *y*) be the direct population flow from location *x* ∈ *X* to location *y* ∈ *X* and *W*(*x*, *x*) = 0 for any *x* ∈ *X*. Based on this, we define an affinity function *A*(*x*, *y*) with

$$\begin{array}{c} A\left(x,y\right)=\frac{W(x,y)+W(y,x)}{2\int_{X}W\left(x,y\right)d\mu \left(x\right)d\mu \left(y\right)} \end{array}$$ (1)

for (*x* ≠ *y*) and *A*(*x*, *y*) = 1 for (*x* = *y*) to quantify the connectivity between location *x* and location *y*. In other words, *A*(*x*, *y*) represents the average of the relative population flow from one location *x* to another *y* and from *y* to *x*.

Our affinity function *A*(*x*, *y*) gives a symmetric and positivity preserving kernel on space *X*. Particularly, *A*(*x*, *y*) would transform the mobility network (MN) into an undirected graph with edge weight *A*(*x*, *y*) if *X* is a set of the nodes of MN. The quantity *P*(*x*, *y*) = *A*(*x*, *y*)/∑_*y*_ *A*(*x*, *y*) can be viewed as the probability for a random walker on *X* to make a step from the vertex *x* to vertex *y*. Naturally, we get the transition matrix *P* and the stationary distribution *c* of this Markov chain given by *c*(*x*) = ∑_*y*_ *A*(*x*, *y*)/∑_*x*,*y*_ *A*(*x*, *y*). Let *P*^(*l*)^(*x*, *y*) be the element of *P*^*l*^ (the powers of matrix *P*) given a scale parameter *l*, we then define the diffusion distance *D*_*l*_(*x*, *y*) between any locations *x* and *y* in space *X* by

$$\begin{array}{c} D_{l}\left(x,y\right)=(\sum_{z}\frac{1}{c(z)}{\left(P^{\left(l\right)}\left(x,z\right)-P^{\left(l\right)}\left(y,z\right)\right)}^{2}{)}^{\frac{1}{2}}, \end{array}$$ (2)

which is a special form of the general diffusion distance [35], and *P*^(*l*)^(*x*, *z*) represents the probability of transition from *x* to *z* in *l* steps. For a sufficiently large *l*, there is a diffusion map [36] that embeds the points {*x* ∈ *X*} into a low-dimensional Euclidean space $R^{k}$ in an approximately isometric manner. That is to say, the diffusion distance of any two points in *X* is approximately equal to the Euclidean distance between the points where they are mapped in $R^{k}$. It is worth noting that Brockmann and Helbing [34] once constructed an effective distance to reduce the complex spatiotemporal spread of infectious disease to a simple wave propagation pattern. Although the effective distance succeeds in giving the pair of points with higher connectivity a smaller distance, the lack of symmetry makes it only a quasi-distance. The diffusion distance (2) in this paper actually defines a metric in space *X*, and *D*_*l*_(*x*, *y*) would also be small if there are many paths connecting *x* and *y*. We use diffusion distance instead of effective distance here to let the involved mathematical operations such as limit and isotropic weighting make sense in this study.

### Reaction diffusion-drift equation

For an epidemic outbreak in the space *X*, we let *p*(*t*, *x*) denote the density of infectious individuals who can move freely and contact others at location *x* ∈ *X* at time *t*. In the perspective of diffusion distance, the evolution of density for infectious individuals can be modeled by a simple reaction-diffusion equation:

$$\begin{array}{c} \frac{\partial p(t,x)}{\partial t}=\gamma \Delta p\left(t,x\right)+R\left(t,x\right)p\left(t,x\right). \end{array}$$ (3)

Eq (3) states that, in each small region, the rate of change in the density of infectious individuals (left-hand side) is balanced by the physical movements with constant diffusion rate *γ* and the net reproduction of infectious individuals. Note that the net growth rate *R*(*t*, *x*) is formulated as *R*(*t*, *x*) = Λ(*t*, *x*) − Γ(*t*, *x*) with the incidence rate Λ(*t*, *x*) and removal rate Γ(*t*, *x*). The incidence rate is the average number of adequate contacts with susceptibles of a typical virus carrier per unit time [37], and is given by

$$\begin{array}{c} \Lambda \left(t,x\right)=\beta C\left(t,x\right)\frac{s(t,x)}{n(t,x)}, \end{array}$$ (4)

where *β* is the transmission probability, *C*(*t*, *x*) is the average contact rate of one person with other individuals, and depends heavily on the intensity of intervention measures. *s*(*t*, *x*) and *n*(*t*, *x*) denote the density of susceptible individuals and the total number of free-moving individuals, respectively.

Facing severe epidemics (e.g., COVID-19), countries around the world have adopted measures such as entry-exit testing and regional lockdown to curb the spatial spread of the pandemic [5]. From a mathematical point of view, this type of interventions introduces a potential function *U* = −*γ* ln(*p*) to counteract diffusion and to maintain a non-uniform density *p*(*t*, *x*) in space *X*. The mobility of infectious individuals is restricted by these interventions since *γ*Δ*p* + ∇.(*p*∇*U*) = 0. The key idea we pursue in this paper is to design a reasonable lockdown exit strategy. To achieve this goal, we introduce another unknown potential function *V* which determines the net travel flux from one location to another in *X*. Combining the function *U* and function *V* together (i.e., *F* = *U* + *V*), we get the complete reaction diffusion-drift equation [38, 39], which has the following form:

$$\begin{array}{c} \frac{\partial p}{\partial t}=\gamma \Delta p+\nabla .(p\nabla F)+Rp. \end{array}$$ (5)

The second term of the Eq (5) represents the spatial movements of individuals induced by the deterministic travel flux −∇*F* in *X*. Obviously, the Eq (5) describes the dynamics of the epidemic under complete lockdown if *V* = 0 (or, ∇*V* = 0), and it would be Eq (3) without any regional lockdown if *V* = *γ* ln(*p*). What we want to do here is to find an optimal *V* to lift the strong lockdown without causing a second outbreak in the epidemic area.

### Lockdown exit strategy

Lifting travel restrictions and restarting travel flux may cause an increase in the contact rate *C*(*t*, *x*) in the selected subregions. We denote *ϵ* to quantify the variation in the contact rate if a new travel flux is initiated. Thus, the net growth rate *R*(*t*, *x*) can be adjusted to a behavior-related growth rate *R*(*t*, *x*, *ϵ*) = Λ(*t*, *x*, *ϵ*) − Γ(*t*, *x*) with

$$\begin{array}{c} \Lambda \left(t,x,\epsilon \right)=\beta ((1-\epsilon )C(t,x)+\epsilon C(0,x))\frac{s(t,x)}{n(t,x)}, \end{array}$$ (6)

We denote *C*(0, *x*) here to represent the normal contact rate at location *x*. Obviously, the lifting of travel restrictions has no effect on the net growth rate if *ϵ* = 0, and the local behavior of individuals has completely returned back to normal if *ϵ* = 1. Although the incidence rate Λ(*t*, *x*) also depends on interventions such as testing, contact tracing and isolation [40], we only introduce an adjustment factor *ϵ* to summarize the variations here without considering the details because the purpose of this paper is to explore the lockdown exit strategy.

A good lockdown exit strategy should first ensure that the relaxation of travel restrictions would not cause a second outbreak of epidemics during the weakening spread stage. In other words, the changing rate in the density of infectious individuals ∂*p*/∂*t* should not be positive after a new deterministic travel flux −∇*V*(*t*_0_, *x*) is initiated at some time *t*_0_. It is summarized by the mathematical expression:

$$\begin{array}{c} \nabla .(p\left(t,x\right)\nabla V\left(t_{0},x\right))+R\left(t,x,\epsilon \right)p\left(t,x\right)\leq 0 \end{array}$$ (7)

for any time *t* ∈ [*t*_0_, *t*_1_), where *t*_1_ is the end time of this travel flux. Here, the diffusion term is cancelled out by the vector field induced by potential function *U*.

#### I. Where and when

Considering the lockdown subregions {Ω_1_, Ω_2_, …, Ω_*m*_} ⊂ *X* and assuming that the growth rate *R*(*t*, *x*, *ϵ*) is homogeneous in each subregion Ω_*i*_(*i* = 1, 2, …, *m*). We integrate the inequality (7) on $\Omega ={⋃}_{i=1}^{m}\Omega_{i}$ and obtain

$$\begin{array}{c} \sum_{i=1}^{m}\int_{\Omega_{i}}\nabla .(p\left(t,x\right)\nabla V\left(t_{0},x\right))d\mu \left(x\right)+\sum_{i=1}^{m}R\left(t,\Omega_{i},\epsilon \right)I\left(t,\Omega_{i}\right)\leq 0, \end{array}$$ (8)

where $I\left(t,\Omega_{i}\right)=\int_{\Omega_{i}}p\left(t,x\right)d\mu \left(x\right)$ is the number of infectious individuals in subregion Ω_*i*_ at time t. Without loss of generality, we assume that the lockdown exit strategy is only implemented among the candidate subregions {Ω_1_, Ω_2_, …, Ω_*m*_}, which means that the infectious individuals moving out from one candidate subregion must enter another one, so the total divergence remains zero. Consequently, $\sum_{i=1}^{m}R\left(t,\Omega_{i},\epsilon \right)I\left(t,\Omega_{i}\right)\leq 0$ is a necessary condition for lifting the strict lockdown in Ω. According to Lemma 1 (S1 Text), the subregions {Ω_1_, Ω_2_, …, Ω_*m*_} satisfying this necessary condition can be selected to initiate some new travel fluxes. We introduce a logical variable *ξ*_*i*_ here to indicate whether the lockdown exit strategy can be implemented in the subregion Ω_*i*_ and obtain the following zero-one programming,

$$\begin{array}{c} \begin{array}{cc} \text{max}\; & H_{1}=\sum_{i=1}^{m}\xi_{i},\\ \text{s}.\text{t}.\; & \sum_{i=1}^{m}R\left(t,\Omega_{i},\epsilon \right)I\left(t,\Omega_{i}\right)\xi_{i}\leq 0,\\ & \xi_{i}\in \left\{0,1\right\}. \end{array} \end{array}$$ (9)

Where, *ξ*_*i*_ = 1 means that the subregion Ω_*i*_ can be selected to initiate a new travel flux at time *t*, and *ξ*_*i*_ = 0 means that the travel restrictions cannot be lifted at this point in time. Maximizing the objective function *H*_1_ to get more subregions where the travel restrictions can be lifted. We can look at the 0 − 1 integer linear programming (9) from two perspectives. On the one hand, we can solve programming (9) at any fixed time *t*_0_ to get the available subregions. On the other hand, we can also focus on the subregions to determine the available time.

#### II. How

Given the available subregions {Ω_1_, Ω_2_, …, Ω_*m*_}, infectious density *p*(*t*, *x*) and growth rate *R*(*t*, *x*, *ϵ*), we hope to determine the potential *V*(*t*_0_, *x*) by solving inequality (7). This is a big challenge, we solve it here based on the approximation theory of diffusion maps [35, 36], one important theory used in this paper. Assuming that the individuals are homogeneous in each subregion, then {Ω_1_, Ω_2_, …, Ω_*m*_} can be regarded as a set of points sampled from the probability density function *f*(*t*_0_, *x*) = *p*(*t*_0_, *x*)/*I*(*t*_0_, *X*). Denote the kernel function

$$\begin{array}{c} K_{\delta }\left(\Omega_{i},\Omega_{j}\right)=\frac{1}{{\left(\pi /\delta \right)}^{k/2}}e^{-\delta D_{l}^{2}\left(\Omega_{i},\Omega_{i}\right)}, \end{array}$$ (10)

where *D*_*l*_(Ω_*i*_, Ω_*i*_) is the diffusion distance (2) between Ω_*i*_ and Ω_*j*_, *k* is the dimension of the embedding space $R^{k}$ corresponding to the diffusion maps [35], and *δ* is a parameter adjusting the kernel width. Based on this kernel, we construct a Laplacian graph *L*_*δ*,*m*_ through the following procedure. Set

$$\begin{array}{c} \begin{array}{cc} & K_{\delta ,m}\left(\Omega_{i},\Omega_{j}\right)=\frac{K_{\delta }\left(\Omega_{i},\Omega_{j}\right)}{\sqrt{\left(\sum_{i=1}^{m}K_{\delta }\left(\Omega_{i},\Omega_{j}\right))(\sum_{j=1}^{m}K_{\delta }\left(\Omega_{i},\Omega_{j}\right)\right)}},\\ & Q_{\delta ,m}\left(\Omega_{i},\Omega_{j}\right)=\frac{K_{\delta ,m}\left(\Omega_{i},\Omega_{j}\right)}{\sum_{j=1}^{m}K_{\delta ,m}\left(\Omega_{i},\Omega_{j}\right)}, \end{array} \end{array}$$

then the graph Laplacian *L*_*δ*,*m*_ = 4*δ*(*Q*_*δ*,*m*_ − *E*_*m*_) converges to the Kolmogorov operator L (Lemma 2 in S1 Text). That is

$$\begin{array}{c} \underset{m\to \infty }{\text{lim}}L_{\delta ,m}=L=\nabla \left(\text{ln}\;p\right)\cdot \nabla +\Delta , \end{array}$$ (11)

where *E*_*m*_ is an identity matrix with dimension *m* × *m*. For the Gaussian kernel, the bandwidth $\sqrt{1/\delta }$ is usually set as the median value of the distances between all samples. Similar to the vector *V*^(*m*)^ = [*V*(*t*_0_, Ω_1_), *V*(*t*_0_, Ω_2_), …, *V*(*t*_0_, Ω_*m*_)]^*T*^, we let *R*^(*m*)^ and *p*^(*m*)^ denote the restriction of functions *R*(*t*_0_, *x*, *ϵ*) and *p*(*t*_0_, *x*) to the candidate subregions, and then construct the following inequality

$$\begin{array}{c} diag(L_{\delta ,m}V^{\left(m\right)}+R^{\left(m\right)})p^{\left(m\right)}\leq 0^{\left(m\right)}, \end{array}$$ (12)

where $diag(\vec{a})$ denotes the diagonal matrix generated by vector $\vec{a}$, and 0^(*m*)^ is a zero vector with dimension *m*. Comparing inequality (12) and (7), the convergence of the Laplacian graph *L*_*δ*,*m*_ (11) implies that any solution *V*^(*m*)^ of inequality (12) is a good approximation to the potential *V* we want to determine, and the error would be very small if the sample sizes *m* are large enough.

We have already mentioned that *V* = 0 means that there is a complete lockdown in the epidemic area *X*, and *V* = *γ* ln(*p*) means that there is no restriction. Our lockdown exit strategy here is to lift travel restrictions as much as possible without worsening the epidemic. Therefore, we give the following optimization to determine *V*^(*m*)^

$$\begin{array}{c} \begin{array}{cc} \text{min}\; & H_{2}={\left\|V^{\left(m\right)}-\gamma \;\text{ln}\left(I^{\left(m\right)}\right)\right\|}^{2},\\ \text{s}.\text{t}.\; & L_{\delta ,m}V^{\left(m\right)}+R^{\left(m\right)}\leq 0^{\left(m\right)}. \end{array} \end{array}$$ (13)

Where *I*^(*m*)^ = [*I*(*t*_0_, Ω_1_), *I*(*t*_0_, Ω_2_), …, *I*(*t*_0_, Ω_*m*_)]^*T*^, and the constraint of optimization (13) is derived from the inequality (12) because *p*^(*m*)^ is positive in epidemic area. Note that we use the number of infectious individuals instead of density in the cost function *H*_2_ because the latter is difficult to quantify in actual data, and the degree of real lockdown is usually adjusted by the size of the number of regional infectious individuals rather than their density. Without loss of generality, we assume that the calculation result yields *V*(*t*_0_, Ω_*i*_) ≥ *V*(*t*_0_, Ω_*j*_) for *i* < *j* because we can always achieve it by adjusting the order of the subregions. The drift process in model (5) is the movement of infectious individuals from the region with high potential to nearby regions with low potential, which leads us to define a net flow matrix ***J*** among the distinct subregions with

$$\begin{array}{c} \begin{array}{c} J_{i,j}=max\{\theta_{i,j}(V\left(t_{0},\Omega_{j}\right)-V\left(t_{0},\Omega_{i}\right))/D_{l}\left(\Omega_{i},\Omega_{i}\right),0\}, \end{array} \end{array}$$ (14)

where the parameter *θ*_*i*,*j*_ ≥ 0 adjusts the net population outflow from subregion Ω_*j*_ to other Ω_*i*_. Consequently, this movement strategy yields a patch model

$$\begin{array}{c} \frac{dI(t,\Omega_{i})}{dt}=\sum_{j=1}^{m}(J_{i,j}I\left(t,\Omega_{j}\right)-J_{j,i}I\left(t,\Omega_{i}\right))+R\left(t,\Omega_{i},\epsilon \right)I\left(t,\Omega_{i}\right) \end{array}$$ (15)

with *i* = 1, …, *m* and *t* ∈ [*t*_0_, *t*_1_). Similar to water flow, the net population movement represented by the matrix ***J*** always moves from subregions with higher potential to subregions with lower potential. For the parameter *θ*_*i*,*j*_, we develop a source-sink method (S1 Text) to determine it. So far our lockdown exit strategy in the lockdown subregions has been quantified by the matrix ***J***. To meet the real needs, we further extend the one-way movement matrix ***J*** to a non-negative matrix ***B*** to present the two-way movements through a population balance equation with random noise

$$\begin{array}{c} B_{i,j}=\left(B_{j,i}-J_{j,i}\right)\frac{I(t,\Omega_{i})}{I(t,\Omega_{j})}+\left(1+\xi \right)J_{i,j}, \end{array}$$ (16)

where the random noise from *ξ* breaks the expected balance in movements. Note that the factor *ξ* should not be less than −1 and *ξ* = 0 means an optimal two-way mobility represented by matrix ***B***. The testable simulations are based on system (15) with the matrix ***B***.

## Real applications

As a verification and test of the method, we apply it to the context of the COVID-19 epidemic with strict control in Mainland China from the end of 2019 to the early part of 2020. We integrate the data from different sources into our method and finally give a movement strategy among the representative cities in Mainland China. More details about calculation and simulation can be found in S1 Text.

### Data

The migration indexes were collected from a website http://qianxi.baidu.com which is an open big data platform including the daily immigration rate and emigration rate of each city in Mainland China. These indices are obtained by dividing the number of people moving out (in) from the current city by the total number of people moving out (in) from all cities in Mainland during the same period. Specifically, a greater migration index in a city is associated with its more outbound (inbound) events by rail, air and road traffic. Although the migration index quantifies the movement of a population, it does not track the movement directions and trajectories of individuals. In other words, the migration index ranks the mobility of population in different cities but does not provide a quantity for the population flow from one city to another. We selected the cities with the top 25 immigration rate or top 25 emigration rate as the objects of our case study.

The population flow from one city to another is derived from other data. Generally, the population flow from one region to another depends on many factors [41] such as the local population size [42, 43], economic level [44] and geographic distance between them [45]. In this study, the per capita Gross Domestic Product (GDP) (corresponding to the local economic level) and the number in the resident population (the local population size) are obtained from the China Statistical Yearbook 2019 [46] and are summarized in S1 Text. The geographic distance between each pair of cities was collected from the Baidu Maps (https://map.baidu.com). Based on these data, we estimated the relative population flow among the selected cities with high migration indices for further analysis.

We obtained the number of daily confirmed COVID-19 cases and the cumulative number of confirmed cases between 23 January and 24 February from the National Health Commission of the People’s Republic of China [47]. Although there will be a time lag in reporting, it is believed that the number of confirmed cases reflect the true epidemic situation in each city.

### Mobility network and diffusion distance

**Fig 1:**
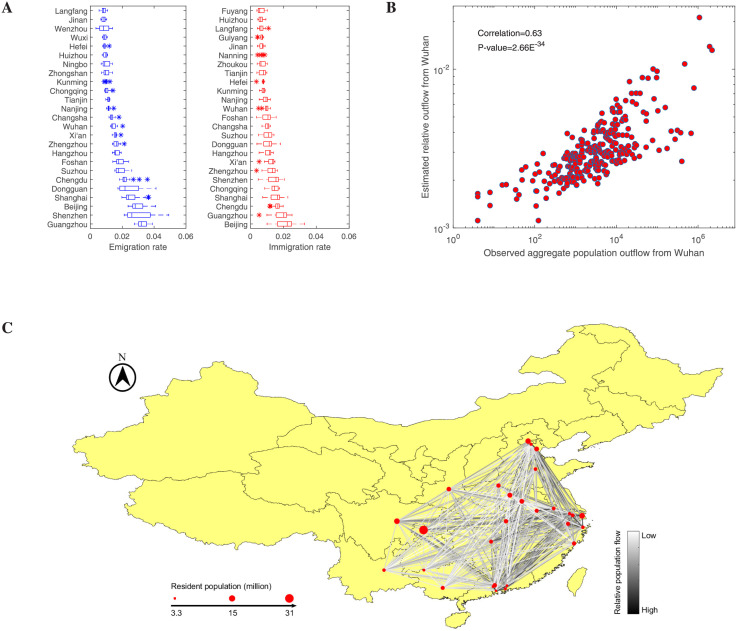
Mobility network among twenty-nine cities with the most active personnel movements in Mainland China in early 2020. **A**: The selected cities are ranked by the value of the migration rate from 1 to 22 January. **B**: The estimated relative outflow from Wuhan to other cities versus the observed aggregate population outflow. **C**: The mobility network among these cities. The diameter of nodes indicates the size of the resident population in each city, and the gray scale indicates the relative population flow between two cities. The base layer of the map was created using public sources: https://data.humdata.org/dataset/china-administrative-boundaries.

We show the distribution of the migration indexes of the selected cities in Fig 1A, and rank these cities according to the mean value of the migration index. These selected cities are mainly divided into labor-intensive export cities with large populations (Chongqing, Zhoukou, Langfang, etc.) or cities with developed economies (Beijing, Shanghai, Guangzhou, Shenzhen, etc.). Due to the Spring Festival, the emigration rate in these cities does appear to be higher than the immigration rate, but the cities ranked by two indices show a high degree of overlap (Fig 1A). Beijing and Guangzhou with the highest emigration rate also had the highest immigration rate. Thus, despite the effect of returning home during the Spring Festival, the population moving out of and into a city still has a weak symmetry. To compensate for the lack of directionality of the migration index, we estimate the direct population flow *W*_*ij*_ from any subregion Ω_*i*_ to subregion Ω_*j*_ (*i* ≠ *j*) with the gravity model [48]

$$\begin{array}{c} W_{ij}=G\frac{M_{i}^{\sigma_{1}}M_{j}^{\sigma_{2}}}{e^{r_{ij}/r}}, \end{array}$$ (17)

where *G* is a proportionality constant, *M*_*i*_ and *M*_*j*_ are the quantitative representation of “mass” (the mean of population size and GDP) in the subregions Ω_*i*_ and Ω_*j*_, respectively. The constant *r* is the characteristic length that governs the decay of population flow with geographic distance *r*_*ij*_. The parameters *σ*_1_ and *σ*_2_ tune the dependence of population flow to the regional mass. The estimation of parameters is derived from a global scale study by Balcan et al. [49].

The statistical results show that the estimated population flow *W*_*ij*_ from region Ω_*i*_ to region Ω_*j*_ and the estimated population flow *W*_*ji*_ from region Ω_*j*_ to region Ω_*i*_ are highly symmetrical (S1 Fig), which is indeed different from the population movement during the Spring Festival. This initially counterintuitive result makes sense when we consider that the population size in each city is always relatively stable and the gravity model is not modeled for a specific period. To further illustrate the reliability of our estimated results, we compared the estimated relative outflow from Wuhan to another 295 cities in China with the real aggregate population outflow (Fig 1B), where the real data come from the work of Jia et al. [2]. The estimated relative outflow from Wuhan show a significant correlation with the real data, and the correlation coefficient is 0.63 with the p-value less than 10^−30^. It is worth noting that the existing data only focus on the population outflow from Wuhan, which can neither form a complete mobility network nor form cross-validation to train the gravity model. Here, we construct an undirected mobility network (MN) with the edges weighted by the relative population flow after symmetrization (1) and visualize it in Fig 1C. The undirected weighted network shows that there were high population flows (the darker edges in Fig 1C) among the cities with the larger population sizes (bigger red dots) and the eastern cities with more developed economies in mainland China. This means that more SARS-CoV-2 carriers came to these cities than to other weakly connected cities before the strict lockdown, which implies that there would be more severe epidemics in these strongly connected cities. Later statistics on the geo-temporal spread of COVID-19 in China [6, 47] fully support this conclusion. In short, it is believed that using the estimated population flow to characterize the connectivity between cities is as reliable as real data.

**Fig 2:**
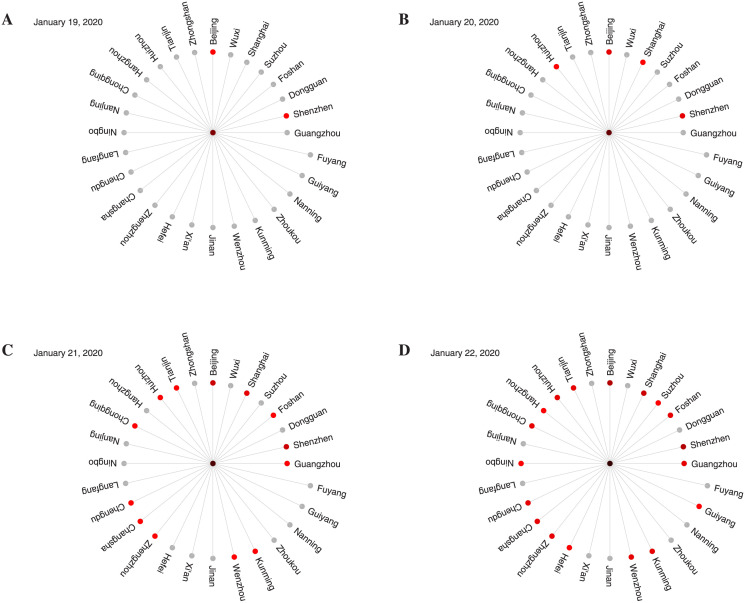
Epidemic invasion trees rooted in Wuhan. The sequence (from 19 to 22 January 2020) of panels depicts the early spread of the SARS-CoV-2 infection in 29 cities. The length of the polar path represents the diffusion distance from Wuhan to each city. The cities with confirmed infected individuals are marked in red, and the prevalence is reflected by the redness of the nodes.

By defining a new diffusion distance (2) derived from the mobility network (MN), we replace the conventional geographic distance by this new metric and visualize the diffusion distances from Wuhan to other selected cities in Fig 2. The length of polar paths connecting Wuhan to other cities in this pandemic invasion tree is the estimated diffusion distance. Interestingly, in the perspective of diffusion distance, Guangzhou is the closest city to Wuhan among the 29 cities, while Fuyang, which has the closest geographical distance to Wuhan, has become the farthest. Moreover, it follows from Fig 2A to 2D that the diffusion distance re-shapes the irregular and complicated spatiotemporal spread patterns of COVID-19 in the conventional geographic perspective [6, 47] into a regular, wavelike solution (i.e., the epidemic first reaches the regions closest to the initial outbreak city). Specifically, COVID-19 cases outside of Hubei Province are first confirmed in Beijing and Shenzhen, and the diffusion distance between Wuhan and them are also shorter than most other cities (Fig 2A). On 20 January 2020, confirmed cases were also reported in Shanghai and Huizhou. Although Shenzhen, Shanghai, Beijing and Huizhou are in the top 11 cities with the smallest diffusion distance from Wuhan (Fig 2B), on 21 and 22 January 2020, COVID-19 cases were confirmed in the cities with larger diffusion distance from Wuhan (Fig 2C and 2D). From this sequence of panels, we find that the shorter the diffusion distance to Wuhan, the earlier the confirmed cases appear, which implies that to a great extent, the diffusion distance can reflect the order of disease spread from the epicenter to other cities. This provides the evidence that our redefined diffusion distance reshapes the spatial spread of the SARS-CoV-2 into a wave-like solution, which led us to connect the spread pattern of COVID-19 with the classical reaction diffusion system [50] in the perspective of diffusion distance.

### Net growth rate

Based on this novel notion of distance, we model the complex spatiotemporal spread patterns of COVID-19 by a simple reaction-diffusion Eq (3) where the diffusion rate *γ* and net growth rate *R* can be estimated from real data. In the early stages of COVID-19 spread, the spatial movements of infected individuals were mainly from the initial outbreak city Ω_0_ to others. Assuming that the infected individuals and net growth rate are homogeneous in each city Ω_*i*_, we get the following ordinary differential equation for the dynamics of the epidemic (details in S1 Text):

$$\begin{array}{c} \frac{dI(t,\Omega_{i})}{dt}=\gamma_{i}\left(t\right)I\left(t,\Omega_{0}\right)+R\left(t,\Omega_{i}\right)I\left(t,\Omega_{i}\right), \end{array}$$ (18)

where *γ*_*i*_(*t*) is the degree of movement from the initial outbreak city Ω_0_ to other cities Ω_*i*_ and assuming that the movement rate *γ*_*i*_(*t*) was constant *γ*_*i*_ (particularly, *γ*_0_ ∝ −*γ*) before lockdown and was 0 during the period of lockdown. In China, the travel ban and the first level response to major public health emergencies were initiated on 23 January 2020 [8], which definitely prevented the movement of population while these measures also reduced the contact rate between people. Therefore, we assume that the incidence rate Λ(*t*, Ω_*i*_) (4) contains an activation function $h\left(t\right)=1/\left(1+e^{t-t_{0}}\right)$ [51] adjusting the contact rate, where *t*_0_ is the critical time point of 23 January 2020. In addition, we can simplify the incidence rate (4) to *βC*(*t*, *x*) since *s*(*t*, *x*) was very close to *n*(*t*, *x*) in the early stages of COVID-19 in China. Consequently, during our research period, the regional net growth rate can be approximated by

$$\begin{array}{c} R\left(t,\Omega_{i}\right)=\Lambda \left(\Omega_{i}\right)h\left(t\right)-\Gamma \left(\Omega_{i}\right), \end{array}$$ (19)

where Λ(Ω_*i*_) is the maximal incidence, Γ(Ω_*i*_) is the removal rate.

Due to reporting delays and infection detection durations, we assume that the daily number of reported new cases $\hat{I}\left(t,\Omega_{i}\right)$ is proportional to the numbers *I*(*t* − *τ*_*i*_, Ω_*i*_) of infectious individuals at *τ*_*i*_ days ago, where the reporting time-delay *τ*_*i*_ is region specific. Therefore, a dynamic system for the daily number of reported new cases is given by

$$\begin{array}{c} \frac{d\hat{I}\left(t,\Omega_{i}\right)}{dt}=\gamma_{i}\left(t-\tau_{i}\right)\hat{I}\left(t-\tau_{i}+\tau_{0},\Omega_{0}\right)+R\left(t-\tau_{i},\Omega_{i}\right)\hat{I}\left(t,\Omega_{i}\right). \end{array}$$ (20)

**Fig 3:**
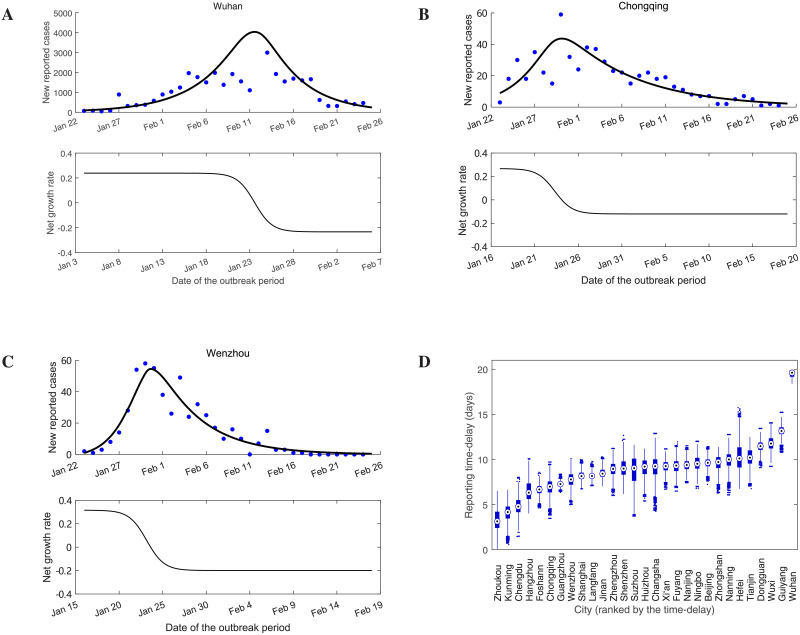
The net growth rates and reporting time delays. **A-C**: The fitted results and the estimated net growth rates (black curves) in three cities with the highest peaks of prevalence in the selected 29 cities. The solid blue dots are real data and one outlier in Wuhan is not shown in the figure. There were different lags between the estimated net growth rates and the numbers of reported cases in each city. **D**: The estimated reporting time delays in all 29 cities. These reporting time delays are region specific.

To this end, we initially estimated the unknown parameters in each city Ω_*i*_ by MCMC methods [52], and then the regional growth rate can be approximated by formula (19) using the estimated parameters. Specifically, we initially fit the model (20) to the daily number of reported new cases in city Ω_0_ and obtain the estimates for parameters (i.e., *γ*_0_, *τ*_0_, Λ(Ω_0_), Γ(Ω_0_), $\hat{I}\left(0,\Omega_{0}\right)$) associated with this city. Secondly, once we have estimates for all parameters about the initial outbreak city Ω_0_, we then apply the same procedure to estimate the relevant parameters (*γ*_*i*_, *τ*_*i*_, Λ(Ω_*i*_), Γ(Ω_*i*_)) and initial value $\hat{I}\left(0,\Omega_{i}\right)$ for each city Ω_*i*_ by fitting model (20) to the daily number of reported new cases in city Ω_*i*_. The mean values of the estimated parameters and their standard deviations are listed in S1 Table. Fig 3A–3C displays the fitted results of the daily number of reported new cases and the estimated net growth rate in three cities (Wuhan, Chongqing and Wenzhou) with the largest peak of confirmed cases in all 29 cities.

The estimated net growth rate *R*(*t*, Ω_*i*_) shows a visible decline in these three cities after 20 January 2020 (Fig 3A–3C), which coincides with fact that on 20 January 2020 confirmation of human-to-human transmission of SARS-CoV-2 was announced and COVID-19 was incorporated as a notifiable disease in the Infectious Disease Law and Health and Quarantine Law in China [53], by which individuals became aware of the risk of disease and started to consciously protect themselves. The net growth rate of these three cities was reduced to zero on about 23 January 2020 (Fig 3A–3C), which indicated that the lockdown of Wuhan and the first-level emergency response in other cities greatly reduced the exposure rate and resulted in a balance between the incidence rate and the removal rate of infected individuals (mainly caused by medical isolation and deaths) in the free moving population. After 23 January, the estimated net growth rate in the three cities decreased to negative values (Fig 3A–3C), indicating that the epidemic curve of new infections in these cities should peak around 23 January 2020, which coincides with the results that the epidemic curve of illness onset peaked around 26 January in China [47] and the median incubation period of Chinese COVID-19 patients was 3 days [54].

It is worth noting that there exists a lag between the time when the net growth rate *R*(*t*, Ω_*i*_) reaches zero and the time when the daily number of reported new cases peaks due to the incubation period of COVID-19 [54, 55] and the delays between diagnosis and case reports. From Fig 3A to 3C, we find that these delays are region specific. Our estimated reporting time delays in all 29 cities are shown in Fig 3D, which illustrates that the delays varied between different cities. This is in agreement with a population-level observational study [56] which showed that there were large differences between Wuhan and other cities in China in terms of reporting time delay. On average, except for Wuhan with a reporting lag of 19 days, the reporting time delays in other cities were less than 15 days, and the numbers of infected individuals in most cities were diagnosed within 10 days.

### Where can be unlocked?

**Fig 4:**
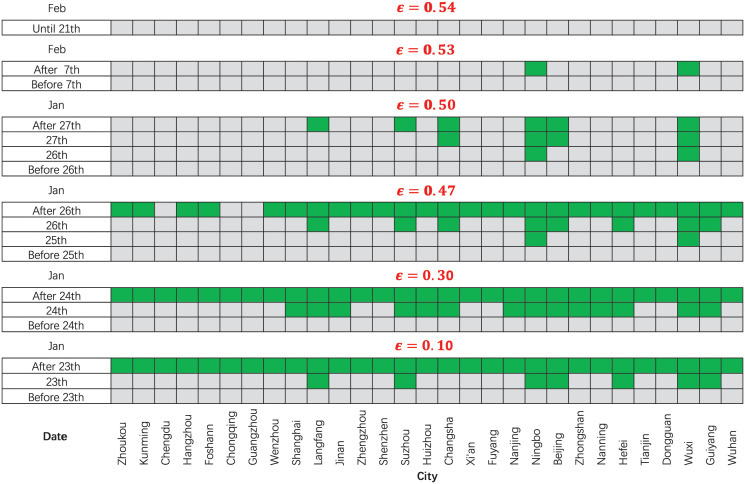
Where and when the lockdown exit strategy can be implemented. With different contact rates, a city is marked green if new travel fluxes can be initiated at that time. The values of *ϵ* quantify the adjustment factor for the contact rate. The larger the value of *ϵ* the higher the contact rate.

To investigate where and when the lockdown exit strategy can be implemented among the selected 29 cities, we solved the optimization problem (9) with various adjustment factors *ϵ* and plot Fig 4 to visualize our calculation results. Note that the adjustment factor *ϵ* quantifies the changes of contact rate after lifting the travel restrictions (6), and the larger the value of *ϵ* the higher the contact rate.

Fig 4 shows that the travel restrictions could not be lifted during the whole study period in all 29 cities if *ϵ* was not less than 0.54. This implies that if the incidence rate Λ(*t*, Ω_*i*_) cannot be maintained at a low level through interventions such as focussing on hygiene, keeping social distancing and maintaining bans on large gatherings, travel restrictions cannot be lifted until the number of infected individuals falls below the local invasion threshold [34]. If the change of contact rate caused by lifting of travel restrictions is relatively small, for example *ϵ* = 0.47 (Fig 4), our calculation results indicate that except for densely populated cities such as Chengdu, Chongqing and Guangzhou, the strict travel restrictions in the other 25 cities could be lifted after 26 January. In particular, for an extremely small value of *ϵ* (for example *ϵ* = 0.1 in Fig 4), travel restrictions in most cities only need to last one day, and it is not necessary to impose strict travel restrictions on the cities such as Langfang, Suzhou, Ningbo, Beijing, Hefei, Wuxi and Guiyang. That is to say, the strict travel restrictions can be lifted early or even be unnecessary in more cities if other mild interventions such as keeping social distance, wearing masks and improving hygiene can maintain the contact rate at a lower level. It is worth noting that the lifting of travel restrictions we are discussing here only implies a non-zero movement rate of the population among the selected cities, not that the individuals can move freely.

### The level of new traffic flow

**Fig 5:**
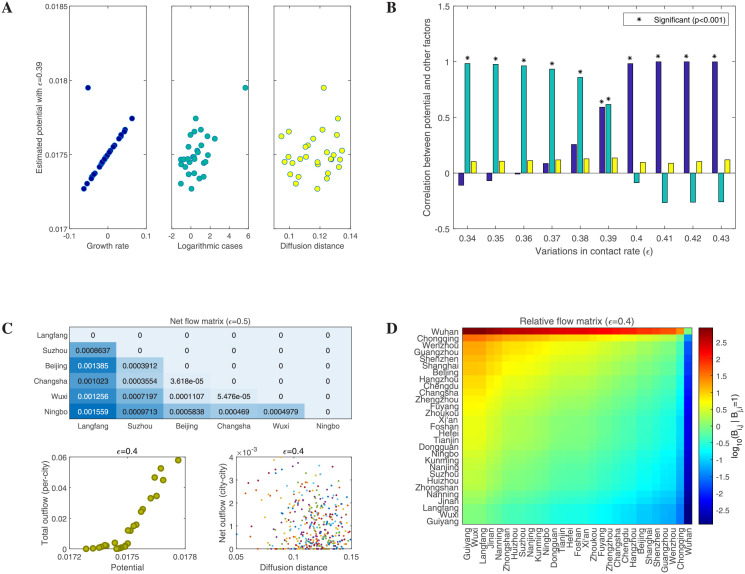
Estimated potential and flow matrices for the selected cities on February 6, 2020. **A**: The estimated potential versus growth rate, COVID-19 cases and diffusion distance. **B**: The correlation between estimated potential and other three factors (i.e., growth rate, COVID-19 cases and diffusion distance) which share the same color used in Fig A. **C**: The reconstructed net flow matrix, and the relationship between outflow and potential and diffusion distance, respectively. The value in each cell (*i*, *j*) of the net flow matrix represents the movement rate from city *j* to city *i*, where *i* is the row label and *j* is the column label. **D**: The relative flow matrix where the value in cell (*i*, *j*) is logarithmic travel flow from city *j* to city *i* under the condition that the travel flow from city *i* to city *j* is 1. The cities are ranked by numbers of COVID-19 cases.

Although we have determined that where and when the lockdown exit strategy can be implemented by solving the optimization problem (9), the details on how to implement the lockdown exit strategy has not been provided yet. In order to obtain the level of optimal traffic flow from one city to another, we first calculate the potential *V*(*t*_0_, Ω_*i*_) for each viable city Ω_*i*_ at available time *t*_0_ by solving the optimization problem (13). Here we calculate potentials for the selected cities on 6 February (two weeks after the lockdown of Wuhan) with different adjustment factors. From the optimization (13), we know that the potential of each city is related to the growth rates and the numbers of infectious individuals in all selected cities, as well as the diffusion distance among them. In Fig 5A, we plot the estimated potential versus the growth rate, the logarithmic number of cases, and the average diffusion distance to other cities. Each of these dots represents a city. We can clearly see that, with an adjustment factor *ϵ* = 0.39, the cities with larger growth rates and more COVID-19 cases tend to have higher potential, but the estimated potential does not show a clear correlation with diffusion distance. We further investigate the correlation between estimated potential and these three factors under different contact rates. From Fig 5B, we find that the estimated potential is significantly correlated with the number of COVID-19 cases when the contact rate is relatively low and is significantly correlated with the growth rate when the contact rate is high. However, there is no significant linear correlation between the potential and diffusion distance. In fact, such results fully meet the requirements of our lockdown exit strategy (13). We hope to remove travel restrictions as much as possible without making the epidemic more severe. This is easier to achieve when the growth rate of COVID-19 cases is lower.

Once we have calculated the potential for each selected city, we define a net flow matrix ***J*** (14) and then approximate the reaction diffusion-drift process (5) into a patch model (15). Fig 4 shows that the lockdown exit strategy can be implemented among Langfang, Suzhou, Beijing, Changsha, Wuxi and Ningbo on 6 February 2020 when *ϵ* = 0.5. We give the net flow matrix among them in Fig 5C. Generally, a city with a higher potential also has more outflows (bottom left corner in Fig 5C), for example, Langfang compared with Ningbo. Although the potential does not show a significant correlation with the diffusion distance, it seems that the outflow population from one city is more likely to reach the closer city (bottom right corner in Fig 5C). Note that the net flow matrix is a lower triangular matrix which means that it only quantifies a one-way net flow of population from the place with high potential to another one with low potential, and cannot reflect the two-way flow of population between the two cities in the actual situation. Here we give a two-way travel strategy through the balance Eq (16) without noise, which shows that the relative movement rate *B*_*i*,*j*_ from city Ω_*j*_ to city Ω_*i*_ can be determined as long as the opposite movement *B*_*j*,*i*_ is given. This provides a quantitative reference for the travel flux, that is, the direct travel flow from one city to another (or vice versa) can be limited to meet the balance equation, which reduces the risk of secondary outbreak of COVID-19 in these cities.

As a special case, we let the travel flow *B*_*j*,*i*_ from city Ω_*i*_ to city Ω_*j*_ be 1, and then calculate the relative travel flow *B*_*i*,*j*_ from city Ω_*j*_ to city Ω_*i*_ according to the balance Eq (16). We visualize the calculated results in Fig 5D where the order of cities is ranked according to the number of COVID-19 cases. The value in each cell (*i*, *j*) of this heatmap is *log*_10_(*B*_*i*,*j*_|*B*_*j*,*i*_ = 1) which is the relative travel flow from city Ω_*j*_ to city Ω_*i*_ after a logarithmic operation. The values in the triangular area above the antidiagonal of the heatmap are positive, while the values below the antidiagonal are negative, and the cells far away from the diagonal are associated with larger absolute values. This indicates that the travel flow from a city with a severe epidemic to a city with a mild epidemic should be smaller than the travel flow in the opposite direction, and the difference between two opposite travel flows increases as the numbers of COVID-19 cases in two cities have a larger difference. It is worth noticing that the relative travel flow between Wuhan and other cities is very different in the two directions (Fig 5D), which means that in order to satisfy the balance Eq (16), the size of the population traveling from Wuhan to other cities should be much less than the size of the population going to Wuhan from other cities. This conclusion is a result of our lockdown exit strategy and is obtained for a specific date, 6 February 2020. Note that our method can calculate the optimal population flows among selected cities at any specific time *t*_0_ as long as this time meets the restrictions of zero-one programming (9).

### Testable predictions

**Fig 6:**
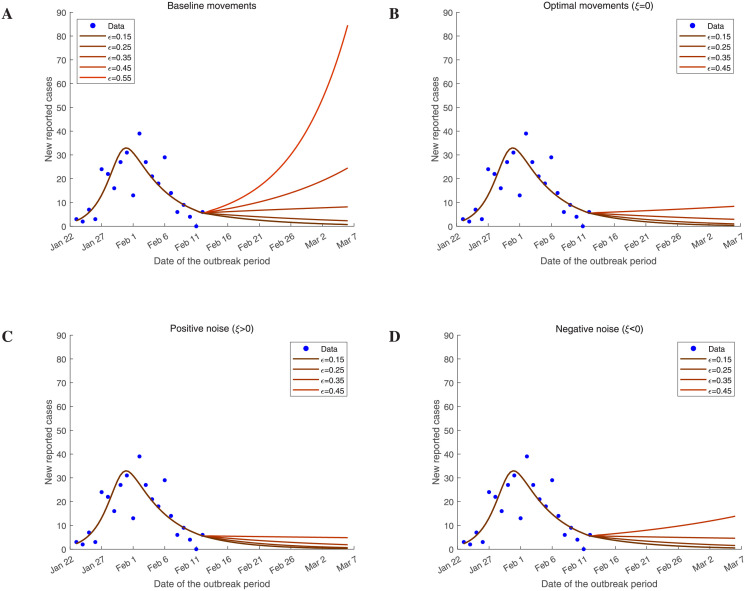
Testable predictions of the number of daily cases reported in Guangzhou under our lockdown exit strategy. **A**: The baseline movements induce a secondary outbreak of COVID-19 in Guangzhou easily. **B**: The travel pattern we present does not cause a secondary outbreak. **C-D**: The simulations based on our travel pattern with positive and negative noise, respectively.

To test our lockdown exit strategy, we ran the patch model (15) with some movement matrices. As a baseline, we first sample from the estimated movement rates (S1 Table) to form a basic movement matrix. Once the basic movement matrix is obtained, we replace the matrix ***J*** in the patch model (15) with it and run model (15) to obtain the predictions of the daily number of reported COVID-19 cases. In Fig 6A, we visualize the simulations in Guangzhou, where the involved movement is a basic movements matrix. In this movements pattern close to the real situation, the epidemic in Guangzhou begins to rebound when the adjustment factor *ϵ* for the contact rate exceeds 0.35 (Fig 6A). On the one hand, it shows that unrestricted movements of the population are likely to cause a second COVID-19 epidemic in Guangzhou. On the other hand, it also reveals that the growth rate plays a core role in the development of the epidemic, and that prevention of the epidemic can also be achieved by reducing the effective contact rate instead of imposing a strict travel ban.

To compare our proposed travel pattern with the baseline movements, we simulate the patch model (15) with movement matrix ***B*** (16). In Fig 6B, we set the interference term *ξ* in the balance Eq (16) to 0, and then simulate the time series of the daily number of reported COVID-19 cases with some acceptable variations in the contact rate. In the first part of our lockdown exit strategy, we already know that this strategy cannot be implemented in Guangzhou if the adjustment factor *ϵ* is not less than 0.47 (Fig 4). We test the acceptable situations and find that the risk of a secondary outbreak of COVID-19 in Guangzhou is greatly reduced according to our calculated movements (Fig 6B). The calculated optimal movements always satisfy a noise-free balance Eq (16) which actually requires the difference between the two-way movements of the population between two cities to be equivalent to the net flow. We further explore what will happen if the balance is broken by some random noise. Here we first consider low noise randomly sampled from the intervals (0, 1) or (−1, 0) as the value of *ξ*. In Fig 6C and 6D, we show the simulations with *ξ* > 0 (we call this positive noise) and *ξ* < 0 (negative noise), respectively. It seems that the positive noise contributes to the extinction of the COVID-19 epidemic in Guangzhou, while the negative noise increases the risk of the second outbreak. This further supports our idea about the lockdown exit strategy, that the population should move from the cities with higher potential to low-potential cities, because the movement matrix ***B*** with positive noise enhances the effect of the net flow matrix ***J*** while negative noise is resisting this pattern.

In order to further verify the effectiveness of the travel scheme that we have designed, we simulate the estimated time series of the daily number of reported new cases for all selected cities in our proposed travel pattern and the baseline travel pattern (S2 Fig). Similar to the simulations in Guangzhou, different travel patterns hardly affect the transmission of COVID-19 in all cities if the value of *ϵ* is relatively small, while with *ϵ* increasing, our proposed travel scheme has notable advantages in preventing the second outbreak of disease in the selected cities. These findings suggest that it is essential for traffic resumption to coincide with reducing the contact rate by wearing masks and maintaining social distancing. Moreover, our designed travel flux pattern will provide a flow scheme to avoid inducing the second outbreak even if the contact rate cannot reach the relatively low level. The simulations in Guangzhou (Fig 6C) show that a moderate increase in the movements flow toward the cities with low potential is beneficial to the prevention and control of the epidemic. However, from S3 Fig we find that if the positive noise is too strong, the movements still increase the number of COVID-19 cases in many cities.

## Conclusion and discussion

With the improvement of the epidemic situation, the prevention and control strategies (lockdown, social distancing and others) in epidemic areas have been adjusted accordingly. In order to avoid the second outbreak of the epidemic, when, where and at what level individuals can flow normally are the critical problems that need to be decided. In this study, we redefined a measure of diffusion distance derived from the underlying mobility network instead of geographic distance and embedded it to a reaction diffusion equation to describe the spread of infectious disease in the population without interventions. We further extend the reaction diffusion model to a reaction diffusion-drift model with diffusion process and drift process of the population. On the basis of the extended spatiotemporal spread model, we developed a novel method to explore when and where and to what level travel flux can be triggered during the late stage of an epidemic using the divergence theorem and diffusion map theory, and under this designed movement pattern among various subregions a second outbreak due to individuals’ movement is impossible.

In the case study, analyzing data on a migration index from 1 to 22 January 2020 gave the top 25 cities with the highest emigration/immigration rates, respectively, and suggested twenty-nine cities with the most movements in mainland China. The mobility network among them is reconstructed from the population size and per capita GDP of each city using a gravity model [48]. We consequently integrated the multi-source data to our method and retrospectively determined the movement matrix of the population among the selected cities at the “correct” time. Specifically, the estimated growth rate and the number of infected individuals determine where and when the lockdown exit strategy can be implemented. The obtained travel flux of populations among the selected cities is actually the net flow of the population moved from a city with high potential to others with low potential. Finally, we test our travel scheme in the selected cities and find that initiating a new traffic flux among these cities according to our designed travel scheme does not cause a second outbreak of COVID-19.

It is worth noting that the case study in this paper is geographically specific, since the data for mainland China used here are more complete and regular than those for other countries, but we hope that the approaches we have used are applicable more generally. In particular, under the condition that the COVID-19 epidemic is still not under full control, all countries have started to resume work and production. Our method provides a possible reasonable travel flux scheme under which individuals’ movements cannot induce the second outbreak. There are still some features and issues about our method to be discussed.

Note that the growth rate of the epidemic and the mobility network of the population are actually input variables in our method, and the estimates of them are part of the preparatory work. The estimation of the growth rate is not limited to a specific method. In the initial outbreak stages where almost all of the population are susceptible (i.e., *S*(*t*)/*N*(*t*) ≈ 1), we simplified the incidence (4) and then fitted the time series of the number of infected individuals using our model to obtain the estimates. For a general community where this simplification is not applicable, the growth rate can also be estimated from epidemic data using the back-tracking method [37].

Lacking available data, we reconstructed the mobility network using a gravity model [48], which takes into account that the links between cities are positively related to their economic activities [57] and population densities [42, 43], but negatively related to the geographic distance [45]. The gravity model provides a feasible approach to estimate the mobility among major cities in mainland China. It is interesting to note that the closer the economic links, the more the high speed trains and flights [58], which has broken the limits of physical distance on mobility. The traditional physical distance is then less significant, hence we adopt the diffusion distance here.

There are three free parameters involved in our method, the transfer step size *l* in the diffusion distance (2), the dimension *k* of the embedding space in the Gaussian kernel (10) and the diffusion rate *γ* in the optimization (13). In the case study, we chose *l* = 25, *k* = 2 and *γ* = 0.03 which is the estimated −*γ*_0_ (S1 Table). We evaluated the variations of these parameters in a relatively large parameter space and found that the changes have little effect on our lockdown exit strategy. Specifically, the change of parameters *k* and *γ* almost has no effect on the minimum-centered potential (S4 Fig), but increasing the parameter *l* reduces it (S4 Fig). Although the variation of *l* affects the value of the estimated potential, the order of estimates has not changed. Cities with higher potential always have higher estimated potential, and the simulations of numbers of COVID-19 cases only rise slightly under extremely large *l* (S4 Fig).

## Supporting information

S1 Fig Outflow versus inflow. The estimated relative population flow among these cities exhibits a high degree of symmetry.
(EPS)

S2 Fig Predictions of reported numbers of daily cases in all cities. The sub-figures in the left column are the simulations in our movement pattern. The sub-pictures in the right column are the simulations in the baseline movement pattern. The simulations in Wuhan are not shown in the graphics window.
(EPS)

S3 Fig Predictions of reported daily cases in all cities. The impact of variations in noise and contact rates on our lockdown exit strategy. The simulations in Wuhan are not shown in the graphics window.
(EPS)

S4 Fig The sensitivity of the estimated potential to three free parameters. **A-C**: The estimated potential function and the variation of potential with changes in the parameters. The variation is the distance between two potential functions with adjacent parameter values. Little variation means that the parameter change has little effect on the calculation of the potential. **D**: Simulations of reported numbers of daily cases in all cities. The adjustment factor *ϵ* = 0.45 and the potential are minimum-centered.
(EPS)

S1 Text Theory and method supplement of this paper. This text provides the computational details and theory supplement for the main text.
(PDF)

S1 Table The estimated values of parameters and initial values. Estimated epidemiological parameters of each city are listed in this table.
(PDF)

S1 Data Data used in the case study. Included in this document are the epidemiological data of each city and the geographic distance between the cities. Demographic data can be found in the S1 Text.
(XLSX)

## References

[1] Wu F, Zhao S, Yu B, Chen Y, Wang W, Song Z, et al. A new coronavirus associated with human respiratory disease in China. Nature. 2020;579(7798):265–269. doi: 10.1038/s41586-020-2008-3 32015508 PMC7094943

[2] Jia JS, Lu X, Yuan Y, Xu G, Jia J, Christakis NA. Population flow drives spatio-temporal distribution of COVID-19 in China. Nature. 2020;582(7798):389–394. doi: 10.1038/s41586-020-2284-y 32349120

[3] Wu JT, Leung K, Leung GM. Nowcasting and forecasting the potential domestic and international spread of the 2019-nCoV outbreak originating in Wuhan, China: a modelling study. The Lancet. 2020;395(10225):689–697. doi: 10.1016/S0140-6736(20)30260-9 32014114 PMC7159271

[4] Tian H, Liu Y, Li Y, Wu CH, Chen B, Kraemer MUG, et al. An investigation of transmission control measures during the first 50 days of the COVID-19 epidemic in China. Science. 2020;368(6491):638–642. doi: 10.1126/science.abb6105 32234804 PMC7164389

[5] Hamzelou J. World in lockdown. New Scientist. 2020;245(3275):7. doi: 10.1016/S0262-4079(20)30611-4 32518458 PMC7270163

[6] Tang B, Wang X, Li Q, Bragazzi NL, Tang S, Xiao Y, et al. Estimation of the transmission risk of the 2019-nCoV and its implication for public health interventions. Journal of Clinical Medicine. 2020;9(2):462. doi: 10.3390/jcm9020462 32046137 PMC7074281

[7] Kraemer MUG, Yang CH, Gutierrez B, Wu CH, Klein B, Pigott DM, et al. The effect of human mobility and control measures on the COVID-19 epidemic in China. Science. 2020;368(6490):493–497. doi: 10.1126/science.abb4218 32213647 PMC7146642

[8] Chinazzi M, Davis JT, Ajelli M, Gioannini C, Litvinova M, Merler S, et al. The effect of travel restrictions on the spread of the 2019 novel coronavirus (COVID-19) outbreak. Science. 2020;368(6489):395–400. doi: 10.1126/science.aba9757 32144116 PMC7164386

[9] Rothstein MA. From SARS to Ebola: legal and ethical considerations for modern quarantine. Indiana Health Law Review. 2015;12:227. doi: 10.18060/18963

[10] Baird RP. What it means to contain and mitigate the Coronavirus; 2020. Website: https://www.newyorker.com/news/news-desk/what-it-means-to-contain-and-mitigate-the-coronavirus.

[11] Cumbria FMD Task Force. Cumbria Foot and Mouth Disease Inquiry Report; 2002. Website: https://cumbria.gov.uk/elibrary/Content/Internet/538/716/37826163827.PDF.

[12] Pearson H, Clarke T, Abbott A, Knight J, Cyranoski D. SARS: what have we learned? Nature. 2003;424(6945):121–126. doi: 10.1038/424121a 12853923 PMC7095391

[13] Fraser C, Donnelly CA, Cauchemez S, Hanage WP, Van Kerkhove MD, Hollingsworth TD, et al. Pandemic potential of a strain of influenza A (H1N1): early findings. Science. 2009;324(5934):1557–1561. doi: 10.1126/science.1176062 19433588 PMC3735127

[14] Nyenswah T, Blackley DJ, Freeman T, Lindblade KA, Arzoaquoi SK, Mott JA, et al. Community quarantine to interrupt Ebola virus transmission-Mawah Village, Bong County, Liberia, August–October, 2014. Morbidity and Mortality Weekly Report. 2015;64(7):179. 25719679 PMC5779591

[15] Badr HS, Du H, Marshall M, Dong E, Squire MM, Gardner LM. Association between mobility patterns and COVID-19 transmission in the USA: a mathematical modelling study. The Lancet Infectious Diseases. 2020;20(11):1247–1254. doi: 10.1016/S1473-3099(20)30553-3 32621869 PMC7329287

[16] Nouvellet P, Bhatia S, Cori A, Ainslie KE, Baguelin M, Bhatt S, et al. Reduction in mobility and COVID-19 transmission. Nature Communications. 2021;12(1):1–9. doi: 10.1038/s41467-021-21358-2 33597546 PMC7889876

[17] Bartik AW, Bertrand M, Cullen Z, Glaeser EL, Luca M, Stanton C. The impact of COVID-19 on small business outcomes and expectations. Proceedings of the National Academy of Sciences. 2020;117(30):17656–17666. doi: 10.1073/pnas.2006991117 32651281 PMC7395529

[18] Bonaccorsi G, Pierri F, Cinelli M, Flori A, Galeazzi A, Porcelli F, et al. Economic and social consequences of human mobility restrictions under COVID-19. Proceedings of the National Academy of Sciences. 2020;117(27):15530–15535. doi: 10.1073/pnas.2007658117 32554604 PMC7355033

[19] Tang S, Xiao Y, Yuan L, Cheke RA, Wu J. Campus quarantine (Fengxiao) for curbing emergent infectious diseases: lessons from mitigating A/H1N1 in Xi’an, China. Journal of Theoretical Biology. 2012;295:47–58. doi: 10.1016/j.jtbi.2011.10.035 22079943

[20] Fraser C, Riley S, Anderson RM, Ferguson NM. Factors that make an infectious disease outbreak controllable. Proceedings of the National Academy of Sciences. 2004;101(16):6146–6151. doi: 10.1073/pnas.0307506101 15071187 PMC395937

[21] Lloyd AL, May RM. Spatial heterogeneity in epidemic models. Journal of Theoretical Biology. 1996;179(1):1–11. doi: 10.1006/jtbi.1996.0042 8733427

[22] Tizzoni M, Bajardi P, Decuyper A, King GKK, Schneider CM, Blondel V, et al. On the use of human mobility proxies for modeling epidemics. PLoS Computational Biology. 2014;10(7):e1003716. doi: 10.1371/journal.pcbi.1003716 25010676 PMC4091706

[23] Ruktanonchai NW, DeLeenheer P, Tatem AJ, Alegana VA, Caughlin TT, zu Erbach-Schoenberg E, et al. Identifying malaria transmission foci for elimination using human mobility data. PLoS Computational Biology. 2016;12(4):e1004846. doi: 10.1371/journal.pcbi.1004846 27043913 PMC4820264

[24] Chang S, Pierson E, Koh PW, Gerardin J, Redbird B, Grusky D, et al. Mobility network models of COVID-19 explain inequities and inform reopening. Nature. 2021;589(7840):82–87. doi: 10.1038/s41586-020-2923-3 33171481

[25] Gilbert M, Dewatripont M, Muraille E, Platteau JP, Goldman M. Preparing for a responsible lockdown exit strategy. Nature Medicine. 2020;26(5):643–644. doi: 10.1038/s41591-020-0871-y 32405055 PMC7155394

[26] Thompson RN, Hollingsworth TD, Isham V, Arribas-Bel D, Ashby B, Britton T, et al. Key questions for modelling COVID-19 exit strategies. Proceedings of the Royal Society B. 2020;287(1932):20201405. doi: 10.1098/rspb.2020.1405 32781946 PMC7575516

[27] Colizza V, Vespignani A. Epidemic modeling in metapopulation systems with heterogeneous coupling pattern: Theory and simulations. Journal of Theoretical Biology. 2008;251(3):450–467. doi: 10.1016/j.jtbi.2007.11.028 18222487

[28] Linka K, Rahman P, Goriely A, Kuhl E. Is it safe to lift COVID-19 travel bans? The Newfoundland story. Computational Mechanics. 2020;66(5):1081–1092. doi: 10.1007/s00466-020-01899-x PMC7456209 32904431

[29] Ruktanonchai NW, Floyd J, Lai S, Ruktanonchai CW, Sadilek A, Rente-Lourenco P, et al. Assessing the impact of coordinated COVID-19 exit strategies across Europe. Science. 2020;369(6510):1465–1470. doi: 10.1126/science.abc5096 32680881 PMC7402626

[30] Tizzoni M, Bajardi P, Poletto C, Ramasco JJ, Balcan D, Gonçalves B, et al. Real-time numerical forecast of global epidemic spreading: case study of 2009 A/H1N1pdm. BMC Medicine. 2012;10(1):1–31. doi: 10.1186/1741-7015-10-165 23237460 PMC3585792

[31] Parino F, Zino L, Porfiri M, Rizzo A. Modelling and predicting the effect of social distancing and travel restrictions on COVID-19 spreading. Journal of the Royal Society Interface. 2021;18(175):20200875. doi: 10.1098/rsif.2020.0875 33561374 PMC8086876

[32] Gösgens M, Hendriks T, Boon M, Steenbakkers W, Heesterbeek H, Van Der Hofstad R, et al. Trade-offs between mobility restrictions and transmission of SARS-CoV-2. Journal of the Royal Society Interface. 2021;18(175):20200936. doi: 10.1098/rsif.2020.0936 33622148 PMC8086858

[33] Noble JV. Geographic and temporal development of plagues. Nature. 1974;250(5469):726–729. doi: 10.1038/250726a0 4606583

[34] Brockmann D, Helbing D. The hidden geometry of complex, network-driven contagion phenomena. Science. 2013;342(6164):1337–1342. doi: 10.1126/science.1245200 24337289

[35] Coifman RR, Lafon S, Lee AB, Maggioni M, Nadler B, Warner F, et al. Geometric diffusions as a tool for harmonic analysis and structure definition of data: Diffusion maps. Proceedings of the National Academy of Sciences. 2005;102(21):7426–7431. doi: 10.1073/pnas.0500334102 15899970 PMC1140422

[36] Coifman RR, Lafon S. Diffusion maps. Applied and Computational Harmonic Analysis. 2006;21(1):5–30. doi: 10.1016/j.acha.2006.04.006 17063683

[37] Zhang J, Lou J, Ma Z, Wu J. A compartmental model for the analysis of SARS transmission patterns and outbreak control measures in China. Applied Mathematics and Computation. 2005;162(2):909–924. doi: 10.1016/j.amc.2003.12.131 32287493 PMC7134600

[38] Ramkrishna D. Population balances: Theory and applications to particulate systems in engineering. Elsevier; 2000.

[39] Weinreb C, Wolock S, Tusi BK, Socolovsky M, Klein AM. Fundamental limits on dynamic inference from single-cell snapshots. Proceedings of the National Academy of Sciences. 2018;115(10):E2467–E2476. doi: 10.1073/pnas.1714723115 29463712 PMC5878004

[40] Wilder B, Charpignon M, Killian JA, Ou HC, Mate A, Jabbari S, et al. Modeling between-population variation in COVID-19 dynamics in Hubei, Lombardy, and New York City. Proceedings of the National Academy of Sciences. 2020;117(41):25904–25910. doi: 10.1073/pnas.2010651117 32973089 PMC7568285

[41] Brockmann D, Hufnagel L, Geisel T. The scaling laws of human travel. Nature. 2006;439(7075):462–465. doi: 10.1038/nature04292 16437114

[42] Zipf GK. The P1 P2/D hypothesis: On the intercity movement of persons. American Sociological Review. 1946;11(6):677–686. doi: 10.2307/2087063

[43] Simini F, Gonzalez MC, Maritan A, Barabasi A. A universal model for mobility and migration patterns. Nature. 2012;484(7392):96–100. doi: 10.1038/nature10856 22367540

[44] Gutierrez J. Location, economic potential and daily accessibility: an analysis of the accessibility impact of the high-speed line Madrid-Barcelona-French border. Journal of Transport Geography. 2001;9(4):229–242. doi: 10.1016/S0966-6923(01)00017-5

[45] Gonzalez MC, Hidalgo ARC, Barabasi A. Understanding individual human mobility patterns. Nature. 2008;453(7196):779–782. doi: 10.1038/nature06958 18528393

[46] National Bureau of Statistics of China. Statistical Yearbook of China 2019. China Statistics Press; 2019.

[47] The Novel Coronavirus Pneumonia Emergency Response Epidemiology Team. The epidemiological characteristics of an outbreak of 2019 novel Coronavirus diseases (COVID-19)-China, 2020. China CDC Weekly. 2020;2(8):113–122. doi: 10.46234/ccdcw2020.032 34594836 PMC8392929

[48] Barthélemy M. Spatial networks. Physics Reports. 2011;499(1-3):1–101. doi: 10.1016/j.physrep.2010.11.002

[49] Balcan D, Colizza V, Gonçalves B, Hu H, Ramasco JJ, Vespignani A. Multiscale mobility networks and the spatial spreading of infectious diseases. Proceedings of the National Academy of Sciences. 2009;106(51):21484–21489. doi: 10.1073/pnas.0906910106 20018697 PMC2793313

[50] Fisher RA. The wave of advance of advantageous genes. Annals of Human Genetics. 1937;7(4):355–369.

[51] Izhikevich EM. Dynamical systems in neuroscience. MIT press; 2007.

[52] Gamerman D, Lopes HF. Markov chain Monte Carlo: stochastic simulation for Bayesian inference. CRC Press; 2006.

[53] Li Q, Guan X, Wu P, Wang X, Zhou L, Tong Y, et al. Early transmission dynamics in Wuhan, China, of novel coronavirus–infected pneumonia. New England Journal of Medicine. 2020;382(13):1199–1207. doi: 10.1056/NEJMoa2001316 31995857 PMC7121484

[54] Guan Wj, Ni Zy, Hu Y, Liang Wh, Ou Cq, He Jx, et al. Clinical Characteristics of Coronavirus disease 2019 in China. New England Journal of Medicine. 2020;382(18):1708–1720. doi: 10.1056/NEJMoa2002032 PMC7092819 32109013

[55] Pan X, Chen D, Xia Y, Wu X, Li T, Ou X, et al. Asymptomatic cases in a family cluster with SARS-CoV-2 infection. Lancet Infectious Diseases. 2020. doi: 10.1016/S1473-3099(20)30114-6 PMC7158985 32087116

[56] Sun K, Chen J, Viboud C. Early epidemiological analysis of the coronavirus disease 2019 outbreak based on crowdsourced data: a population-level observational study. The Lancet Digital Health. 2020;2(4):e201–e208. doi: 10.1016/S2589-7500(20)30026-1 32309796 PMC7158945

[57] Dong L, Chen S, Cheng Y, Wu Z, Li C, Wu H. Measuring economic activity in China with mobile big data. EPJ Data Science. 2017;6:1–17.32355601

[58] Knipl DH, Rost G, Wu J. Epidemic spread and variation of peak times in connected regions due to travel-related infections dynamics of an antigravity-type delay differential model. SIAM Journal on Applied Dynamical Systems. 2013;12(4):1722–1762. doi: 10.1137/130914127

