# Supplementary material for: Determining travel fluxes in epidemic areas
Source: PLoS Comput Biol. 2021 Oct 27;17(10):e1009473. doi: 10.1371/journal.pcbi.1009473 (PMC8550429; doi:10.1371/journal.pcbi.1009473)
Supplement: S1 Text — This text provides the computational details and theory supplement for the main text. (PDF) [file pcbi.1009473.s005.pdf]

Computational details for the main text

0.1 Data preparation for gravity model

The gravity model that quantifies the population flow between any two cities is inspired by Newton's law of gravity. The resident population and GDP per capita in each city can be regarded as the "mass" of each city. The demographic data used in this study are listed in following table.

Table 1: The resident population and GDP per capita in selected cities. Note: GDP (CNY), population (ten thousand). Source: Statistical Yearbook of China 2019 [1].

**City**	Wuhan	Beijing	Chengdu	Dongguan	Foshan	Fuyang	Guangzhou	Guiyang	Hangzhou
**GDP/capita**	135136	140211	94782	98939	127691	21589	155491	78449	140180
**Population**	1108.1	2154.2	1633	839.22	790.57	820.7	1490.44	488.19	980.6
**City**	Hefei	Huizhou	Jinan	Kunming	Langfang	Nanjing	Nanning	Ningbo	Shanghai
**GDP/capita**	97470	85418	106302	76387	64906	152886	60626	132603	134982
**Population**	808.7	483	746.04	685	483.66	843.62	725.41	820.2	2423.78
**City**	Shenzhen	Suzhou	Tianjin	Wenzhou	Wuxi	Xi'an	Changsha	Zhengzhou	Zhongshan
**GDP/capita**	189568	173765	120710	72657	174270	85114	134933	101349	110585
**Population**	1302.66	1072.17	1559.6	925	657.45	1000.37	815.47	1013.6	331
**City**	Chongqing	Zhoukou							
**GDP/capita**	65933	30817							
**Population**	3101.79	867.78							

Before applying the gravity model in main text, we first adjust the data to the same order of magnitude. Here we reduce the value of GDP by 100 times, and then use the

mean of it and population size as the "mass" of each city. That is

$$M_i = \frac{1}{2}(GDP_i/100 + Population_i), \tag{A.1}$$

where GDP_i and $Population_i$ are the GDP per capita and resident population in city Ω_i, respectively.

0.2 The ODE models for parameter estimation

Assume that the infected individuals and net growth rate are homogeneous in each city Ω_i, we integrate the original model (3) on the initial outbreak city Ω_0, and get

$$\frac{dI(t,\Omega_0)}{dt} = \gamma \int_{\Omega_0} \Delta p(t,x)dx + R(t,\Omega_0)I(t,\Omega_0). \tag{A.2}$$

The divergence theorem derives

$$\int_{\Omega_0} \Delta p(t,x)dx = \int_{\partial\Omega_0} \nabla p(t,x) \cdot d\boldsymbol{S}(x). \tag{A.3}$$

Note that the right term in this equation is not well defined before we introduce a Dirac vector function $\boldsymbol{\delta}(x)$ [2] on the boundary $\partial\Omega_0$. With this Dirac vector function, the gradient of $p(t,x)$ on the discontinuous boundary $\partial\Omega_0$ is

$$\nabla p(t,x) = \boldsymbol{\delta}(x)(p(t,\Omega_0^{outside}) - p(t,\Omega_0^{inside})). \tag{A.4}$$

In the early stages of the COVID-19 outbreak in China, the epidemic in Wuhan is far worse than other cities. Thus, we approximate the gradient as

$$\nabla p(t,x) = -\boldsymbol{\delta}(x)I(t,\Omega_0)/|\Omega_0|. \tag{A.5}$$

Consequently,

$$\int_{\Omega_0} \Delta p(t,x)dx = -\frac{\int_{\partial\Omega_0} \boldsymbol{\delta}(x)\cdot d\boldsymbol{S}(x)}{|\Omega_0|} I(t,\Omega_0) = -\frac{|\partial\Omega_0|}{|\Omega_0|} I(t,\Omega_0). \tag{A.6}$$

Therefore, we get a ODE model for the epidemic in the city Ω_0

$$\frac{dI(t,\Omega_0)}{dt} = \gamma_0 I(t,\Omega_0) + R(t,\Omega_0)I(t,\Omega_0), \tag{A.7}$$

where $\gamma_0 = -|\partial\Omega_0|/|\Omega_0|\gamma$ is the total outflow rate from the initial outbreak city Ω_0.

Before the lockdown in Wuhan, the infected individuals mainly spread from Wuhan to other cities. We generalize the ODE model for city Ω_0 to a general form for each city Ω_i, yields

$$\frac{dI(t,\Omega_i)}{dt} = \gamma_i I(t,\Omega_0) + R(t,\Omega_i)I(t,\Omega_i), \tag{A.8}$$

where γ_i is the movement rate of population form the initial outbreak city Ω_0 to another city Ω_i.

0.3 Source-sink method

Based on the behavior-related growth rate $R(t_0,\Omega_i,\epsilon)$, we distinguish the subregions into source subregion with $R(t_0,\Omega_i,\epsilon) > 0$ and sink subregion with $R(t_0,\Omega_i,\epsilon) < 0$. After getting the estimated potential $V(\Omega_1) > V(\Omega_2) > ... > V(\Omega_m)$, we first give a basic matrix J with the element

$$J_{i,j} = max\big\{\big(V(\Omega_j) - V(\Omega_i)\big)/D(\Omega_i,\Omega_j), 0\big\}, \tag{A.9}$$

where $D(\Omega_i, \Omega_j)$ is the diffusion distance between Ω_i and Ω_j. And then, we test the patch model (15) using this matrix J as a mobility matrix. For every $i = 1, ..., m$, we calculate the right term of patch model (15). For

$$\sum_{j=1}^{m} \big(J_{i,j} I(t_0, \Omega_j) - J_{j,i} I(t_0, \Omega_i)\big) + R(t_0, \Omega_i, \epsilon) I(t_0, \Omega_i) > threshold, \tag{A.10}$$

we orderly set $\bar{J}_{i,j} = 0$ $(j = 1, ..., m)$ if Ω_j is a sink subregion until the left term of the inequality (A.10) is non-positive or all sink subregions have been traversed, and $\bar{J}_{i,j} = J_{i,j}$ otherwise. In this way, we reduce the risk of a short-term outbreak of the epidemic in subregion Ω_i due to the population flow from the sink subregion Ω_j. To further reduce the rebound probability of infectious diseases, we traverse source subregions and repeat the above process. Larger *threshold* in (A.10) means higher population flow following the gradient of potential V. We set $threshold = 0.1$ and introduce noise into the balance equation (16) in the main text to discuss the effect of its changes. The positive noise in main text has a similar effect on population flow matrix.

With the updated matrix $\bar{J} = \{\bar{J}_{i,j}\}$, we calculate the right term of patch model (15) again. For every $i = 1, ..., m$, if

$$\sum_{j=1}^{m} \big(\bar{J}_{i,j} I(t_0, \Omega_j) - \bar{J}_{j,i} I(t_0, \Omega_i)\big) + R(t_0, \Omega_i, \epsilon) I(t_0, \Omega_i) < 0, \tag{A.11}$$

we denote

$$\vartheta_i = \frac{\sum_{j=1}^{m} \bar{J}_{j,i} I(t_0, \Omega_i) - R(t_0, \Omega_i, \epsilon) I(t_0, \Omega_i)}{\sum_{j=1}^{m} \bar{J}_{i,j} I(t_0, \Omega_j) + eps}, \tag{A.12}$$

where $eps > 0$ is small enough to ensure that the denominator is not zero. And then, we

update

$$\tilde{J}_{i,j} = \min\{\vartheta_i \bar{J}_{i,j}, J_{i,j}\}. \tag{A.13}$$

In this process, we promote the movement of population to subregions with low potential, while ensuring that the elements of the mobility matrix do not exceed the basic matrix to avoid population gathering in specific areas.

0.4 Theory Supplement

Here, we list the theoretical results used to determine the available subregions and travel fluxes in epidemic areas.

Lemma 1. For any continuous (piecewise continuous) function $g(x)$ with $\int_\Omega g(x)d\mu(x) \leq 0$ in the measure space $(X, \mathcal{A}, \mu)$, there is a integrable function $h(x)$ with $\int_\Omega h(x)d\mu(x) = 0$, such that $g(x) + h(x) \leq 0$ for any $x \in \Omega \subset X$.

Proof. We decompose the function $g(x)$ by

$$g(x) = g_+(x) + g_-(x)$$

with $g_+(x) \geq 0$ and $g_-(x) \leq 0$.

Let

$$A = \int_\Omega g_+(x)d\mu(x), \quad B = \int_\Omega g_-(x)d\mu(x).$$

If $B = 0$ (i.e., $g_-(x) = 0$), then $g(x) = 0$. We take $h(x) = 0$.

If $B < 0$, then $\frac{A}{B} + 1 \geq 0$. We take $h(x) = \frac{A}{B}g_-(x) - g_+(x)$. □

Lemma 2 (Special case of Theorem 2 from Coifman et al. [3]). Assume that the data set $\{x_1, x_2, ..., x_m\}$ are obtained from a probability density $f(x)$ supported on manifold $\Omega \subset R^k$. Given a rotation-invariant kernel $k_\delta(x,y) = \left(\frac{\delta}{\pi}\right)^{k/2} e^{-\delta\|x-y\|^2}$ for points x and y. Set $b_\delta(x_i) = \sum_{j=1}^m k_\delta(x_i, x_j)$, $k_{\alpha,\delta}(x_i, x_j) = \frac{k_\delta(x_i,x_j)}{b_\delta^\alpha(x_i) b_\delta^\alpha(x_j)}$, $d_{\alpha,\delta}(x_i) = \sum_{j=1}^m k_{\alpha,\delta}(x_i, x_j)$, and $P_{\alpha,\delta}(x_i, y_j) = \frac{k_{\alpha,\delta}(x_i,x_j)}{d_{\alpha,\delta}(x_i)}$ is the transition kernel of matrix P_m, then the graph Laplacian $L_{\delta,m} = 4\delta(P_m - E)$ converges to a infinitesimal generator $\mathcal{L}_\alpha$ of the diffusion, i.e., $\lim_{\delta,m\to\infty} L_{\delta,m}\phi = \mathcal{L}_\alpha \phi = 2(1-\alpha)\nabla(\ln f)\cdot\nabla\phi + \Delta\phi$ for any smooth function $\phi \in C^2(\Omega)$.

Proof. When the sample size m are large enough, the law of large numbers yeilds

$$b_\delta(x) = \lim_{m\to\infty} \sum_{j=1}^m k_\delta(x, x_j) = \int_\Omega k_\delta(x,y) f(y) dy.$$

Using the Taylor expansion of $f(y)$, we have

$$b_\delta(x) - f(x) = \int_\Omega k_\delta(x,y)\Big((y-x)\nabla f + \frac{1}{2}(y-x)H(y-x)^T + O(\|y-x\|^3)\Big)dy,$$

where $\nabla f = (\frac{\partial f}{\partial x_1}, \frac{\partial f}{\partial x_2}, ..., \frac{\partial f}{\partial x_k})^T$ is the gradient of f and H is the corresponding Hessian.

First, for $i \neq j$,

$$\int_\Omega k_\delta(x,y)(y-x)_i dy = 0, \quad \int_\Omega k_\delta(x,y)(y-x)_i(y-x)_j dy = 0. \tag{A.14}$$

Second, for $i = j$,

$$\int_\Omega k_\delta(x,y)(y-x)_i^2 dy = \left(\frac{\delta}{\pi}\right)^{k/2} \int_\Omega (y-x)_i^2 e^{-\delta\|x-y\|^2} dy = \frac{1}{2\delta}. \tag{A.15}$$

Further, we have

$$\int_\Omega k_\delta(x,y)\|y-x\|^3 dy = \left(\frac{\delta}{\pi}\right)^{k/2} \int_\Omega \|y-x\|^3 e^{-\delta\|x-y\|^2} dy = O(\delta^{-\frac{3}{2}}). \tag{A.16}$$

These equations (A.14-A.16) immediately imply that

$$\begin{aligned} b_\delta(x) = \int_\Omega k_\delta(x,y)f(y)dy &= f(x) + \frac{1}{4\delta}\sum_{i=1}^{k}\frac{\partial^2 f(x)}{\partial^2 x_i} + o(\frac{1}{\delta}) \\ &= f(x) + \frac{1}{4\delta}\Delta f(x) + o(\frac{1}{\delta}). \end{aligned} \tag{A.17}$$

Similarly, we can calculate $d_{\alpha,\delta}(x)$ by

$$\begin{aligned} d_{\alpha,\delta}(x) &= \lim_{m\to\infty}\sum_{j=1}^{m} k_{\alpha,\delta}(x_i,x_j) = \int_\Omega k_{\alpha,\delta}(x,y)f(y)dy \\ &= \frac{1}{b_\delta^\alpha(x)f^{1-\alpha}(x)}\left[1 + \frac{1}{4\delta}\left(\frac{\Delta(f^{1-\alpha}(x))}{f^{1-\alpha}(x)} - \alpha\frac{\Delta f(x)}{f(x)}\right) + o(\frac{1}{\delta})\right]. \end{aligned} \tag{A.18}$$

Consequently, the shift operator $P_{\alpha,\delta}$, which is the limitation of the transition matrix P_m for infinite samples, has the asymptotic expansion as

$$\begin{aligned} P_{\alpha,\delta}\phi(x) &= \frac{1}{d_{\alpha,\delta}(x)}\int_\Omega k_{\alpha,\delta}(x,y)\phi(y)f(y)dy \\ &= \phi(x) + \frac{1}{4\delta}\left[\frac{\Delta(\phi(x)f^{1-\alpha}(x))}{f^{1-\alpha}(x)} - \phi(x)\frac{\Delta f^{1-\alpha}(x)}{f^{1-\alpha}(x)}\right] + o(\frac{1}{\delta}) \end{aligned}$$

since

$$\int_\Omega k_{\alpha,\delta}(x,y)\phi(y)f(y)dy = \frac{1}{b_\delta^\alpha(x)f^{1-\alpha}(x)}\left[\phi(x)+\frac{1}{4\delta}\left(\frac{\Delta(\phi(x)f^{1-\alpha}(x))}{f^{1-\alpha}(x)}-\alpha\phi(x)\frac{\Delta f(x)}{f(x)}\right)+o(\frac{1}{\delta})\right].$$

Therefore, we have the infinitesimal generator $\mathcal{L}_\alpha$ of the diffusion as

$$\mathcal{L}_\alpha\phi = \lim_{\delta\to\infty} 4\delta(P_{\alpha,\delta} - E)\phi = \frac{\Delta(\phi f^{1-\alpha})}{f^{1-\alpha}} - \frac{\Delta(f^{1-\alpha})}{f^{1-\alpha}}\phi = 2(1-\alpha)\nabla(\ln f)\cdot\nabla\phi + \Delta\phi.$$

□

References

[1] National Bureau of Statistics of China, *Statistical Yearbook of China 2019*, (China Statistics Press, 2019).

[2] I. M. Gel'fand, N. Y. Vilenkin, *Generalized functions: applications of harmonic analysis*, vol. 4 (Academic Press, 2014).

[3] R. R. Coifman, S. Lafon, *Applied and computational harmonic analysis* **21**, 5 (2006).
