# Supplementary material for: Determining travel fluxes in epidemic areas
Source: PLoS Comput Biol. 2021 Oct 27;17(10):e1009473. doi: 10.1371/journal.pcbi.1009473 (PMC8550429; doi:10.1371/journal.pcbi.1009473)
Supplement: S1 Table — Estimated epidemiological parameters of each city are listed in this table. (PDF) [file pcbi.1009473.s006.pdf]

**Parameter**	$\Lambda(\Omega_i)$ / Mean (std)	$\Gamma(\Omega_i)$ / Mean (std)	τ_i / Mean (std)	γ_i / Mean (std)	$\hat{I}(0,\Omega_i)$
**Description**	Maximal incidence	Removal rate	Reporting delay	Inflow from Wuhan	Initial value
Wuhan	0.482 (0.003)	0.238 (0.007)	19.500 (0.121)	-0.030 $(1.804 * 10^{-4})$	87.175 (7.034)
Beijing	0.370 (0.079)	0.182 (0.014)	9.732 (0.856)	$7.868 * 10^{-5}$ $(1.477 * 10^{-4})$	6.293 (2.178)
Chengdu	0.492 (0.019)	0.142 (0.026)	4.693 (1.345)	$2.028 * 10^{-4}$ $(2.264 * 10^{-4})$	2.145 (0.883)
Dongguan	0.455 (0.013)	0.180 (0.011)	11.461 (0.856)	$6.241 * 10^{-5}$ $(3.58 * 10^{-5})$	0.284 (0.583)
Foshan	0.516 (0.041)	0.154 (0.019)	5.718 (1.035)	$6.664 * 10^{-5}$ $(6.642 * 10^{-5})$	0.958 (0.697)
Fuyang	0.507 (0.011)	0.189 (0.008)	9.423 (0.557)	$4.226 * 10^{-5}$ $(3.337 * 10^{-5})$	0.858 (0.608)
Guangzhou	0.510 (0.059)	0.161 (0.016)	7.045 (0.522)	$2.581 * 10^{-4}$ $(2.67 * 10^{-4})$	2.314 (1.187)
Guiyang	0.513 (0.003)	0.205 (0.001)	12.762 (1.276)	$1.860 * 10^{-5}$ $(1.094 * 10^{-5})$	0.052 (0.576)
Hangzhou	0.477 (0.027)	0.167 (0.020)	5.483 (0.596)	$2.012 * 10^{-5}$ $(1.303 * 10^{-4})$	2.835 (0.922)
Hefei	0.459 (0.040)	0.208 (0.013)	10.590 (1.319)	$3.997 * 10^{-5}$ $(5.501 * 10^{-5})$	1.404 (0.911)
Huizhou	0.517 (0.025)	0.181 (0.003)	8.546 (0.942)	$2.434 * 10^{-5}$ $(2.135 * 10^{-5})$	0.395 (0.592)
Jinan	0.522 (0.012)	0.208 (0.007)	7.756 (1.220)	$3.538 * 10^{-5}$ $(3.226 * 10^{-5})$	0.456 (0.612)
Kunming	0.500 (0.035)	0.155 (0.020)	4.556 (1.150)	$1.079 * 10^{-4}$ $(1.337 * 10^{-4})$	0.782 (0.730)
Langfang	0.471 (0.009)	0.197 (0.003)	7.650 (2.443)	$4.388 * 10^{-4}$ $(7.924 * 10^{-5})$	0.309 (0.598)
Nanjing	0.500 (0.010)	0.204 (0.005)	9.636 (0.690)	$3.443 * 10^{-4}(3.251 * 10^{-5})$	0.597 (0.602)
Nanning	0.478 (0.007)	0.206 (0.006)	10.219 (0.906)	$3.551 * 10^{-5}$ $(2.510 * 10^{-5})$	0.306 (0.588)
Ningbo	0.434 (0.018)	0.228 (0.014)	9.725 (0.548)	$2.103 * 10^{-4}(1.115 * 10^{-4})$	1.419 (0.805)
Shanghai	0.407 (0.026)	0.170 (0.009)	7.561 (0.655)	$9.118 * 10^{-5}$ $(7.678 * 10^{-5})$	5.589 (1.074)
Shenzhen	0.481 (0.018)	0.200 (0.007)	8.264 (0.380)	$6.091 * 10^{-4}$ $(2.349 * 10^{-4})$	1.884 (1.113)
Suzhou	0.451 (0.012)	0.207 (0.018)	8.510 (1.061)	$9.565 * 10^{-5}$ $(5.640 * 10^{-5})$	0.963 (0.653)
Tianjin	0.503 (0.014)	0.196 (0.005)	10.768 (0.699)	$2.081 * 10^{-5}(2.075 * 10^{-5})$	0.580 (0.598)
Wenzhou	0.515 (0.014)	0.199 (0.016)	7.156 (0.340)	0.001 $(1.930 * 10^{-4})$	0.968 (0.784)
Wuxi	0.470 (0.010)	0.234 (0.005)	11.847 (0.794)	$2.750 * 10^{-5}$ $(1.976 * 10^{-5})$	0.344 (0.586)
Xi'an	0.475 (0.023)	0.191 (0.009)	9.407 (0.898)	$5.005 * 10^{-5}$ $(4.592 * 10^{-5})$	0.856 (0.646)
Changsha	0.395 (0.013)	0.195 (0.020)	9.079 (0.698)	$2.469 * 10^{-4}(1.419 * 10^{-4})$	2.932 (0.934)
Zhengzhou	0.469 (0.023)	0.176 (0.038)	7.868 (1.955)	$6.269 * 10^{-5}$ $(2.590 * 10^{-4})$	1.639 (0.922)
Zhongshan	0.494 (0.015)	0.198 (0.004)	9.983 (0.785)	$3.465 * 10^{-5}$ $(2.967 * 10^{-5})$	0.330 (0.590)
Chongqing	0.387 (0.046)	0.120 (0.013)	6.304 (0.737)	$1.725 * 10^{-5}$ $(2.384 * 10^{-4})$	8.923 (1.993)
Zhoukou	0.535 (0.033)	0.143 (0.007)	4.327 (0.783)	$2.036 * 10^{-4}$ $(1.047 * 10^{-4})$	0.509 (0.779)
